# Reliable Denoising Strategy to Enhance the Accuracy of Arrival Time Picking of Noisy Microseismic Recordings

**DOI:** 10.3390/s23239421

**Published:** 2023-11-26

**Authors:** Xiaohui Zhang, Huailiang Li, Wenzheng Rong

**Affiliations:** 1 Key Laboratory of Earth Exploration and Information Technology, Chengdu University of Technology, Ministry of Education, Chengdu 610059, China; zhangxiaohui@stu.cdut.edu.cn (X.Z.); rwz0910@126.com (W.R.); 2 State Key Laboratory of Geohazard Prevention and Geoenvironment Protection, Chengdu University of Technology, Chengdu 610059, China

**Keywords:** microseismic data, first arrival picking, ensemble empirical mode decomposition, sample entropy

## Abstract

We propose a method to enhance the accuracy of arrival time picking of noisy microseismic recordings. A series of intrinsic mode functions (IMFs) of the microseismic signal are initially decomposed by employing the ensemble empirical mode decomposition. Subsequently, the sample entropy values of the obtained IMFs are calculated and applied to set an appropriate threshold for selecting IMFs. These are then reconstructed to distinguish between noise and useful signals. Ultimately, the Akaike information criterion picker is used to determine the arrival time of the denoised signal. Test results using synthetic noisy microseismic recordings demonstrate that the proposed approach can significantly reduce picking errors, with errors within the range of 1–3 sample intervals. The proposed method can also give a more stable picking result when applied to different microseismic recordings with different signal-to-noise ratios. Further application in real microseismic recordings confirms that the developed method can estimate an accurate arrival time of noisy microseismic recordings.

## 1. Introduction

As a reliable and effective monitoring tool, the microseismic method has been extensively used in various domains such as rock bursts, hydraulic fracturing monitoring, or mining safety [1,2,3]. Precise arrival time identification is crucial to obtaining an accurate microseismic location result. High-quality estimates of arrival times can significantly enhance the efficiency of the microseismic monitoring process [4,5]. Currently, numerous well-establish pickers are available for evaluating the arrival times of high-quality microseismic recordings, including the autoregressive Akaike information criterion (AIC) and various improved pickers [6,7,8,9], short/long time averaging (STA/LTA) [10], modified energy ratio (MER) [11,12], modified Coppens’ method (MCM) [13,14], and higher-order statistics methods which involving skewness and kurtosis algorithms [15,16]. Additionally, various reliable approaches are designed to suppress the noise in noisy microseismic recordings and develop joint picking methods. The filter algorithms are primarily based on the popular linear digital filter, polarization filter, wavelet transform, etc. [17,18,19,20]. Recently, some new approaches based on deep learning have been proposed to achieve reliable arrival time estimations, which benefit from the modern computer platform with high-performance [21,22,23]. All the aforementioned picking approaches have made significant contributions to enhancing the precision of arrival time identification in different historical periods, and several algorithms remain the most widely used nowadays.

However, digital filters usually need to set different parameters based on the different principal frequencies of the microseismic data [24,25]. Polarization filters need to set a reasonable sliding time window combining with the principal frequency of microseismic signals [26,27]. Moreover, predicting the corresponding principal frequency in microseismic recordings with high noise is difficult. Similarly, wavelet transforms also need to set different parameters according to the different characteristics of microseismic data, such as wavelet basis, wavelet decomposition level, etc. [28,29]. Traditional machine learning methods depend strongly on some hand-engineered features of the sample dataset, requiring training predictive models through analyzing these crucial features [30,31]. Nevertheless, obtaining some apparent quantitative features from microseismic data might be challenging. As the most representative branch of machine learning algorithms, deep learning can construct reliable prediction models using some crucial features extracted through self-learning. However, deep learning relies heavily on well-designed training datasets, and each sample trajectory should contain one or more effective microseismic events in the training dataset [32,33]. Moreover, deep learning involves substantial computational complexity, requiring corresponding high-performance computing platforms.

Motivated by the aforementioned facts and taking into account the noisy environments encountered during in-situ monitoring as well as the intrinsic non-linearity and non-stationary nature of microseismic signals, the extraction of undistorted, useful signals from high-noise microseismic data, followed by the application of a robust picker, emerges as a reasonable approach. In this study, we introduce a reliable denoising algorithm employing sample entropy (SampEn) and ensemble empirical mode decomposition (EEMD), capable of obtaining high-quality undistorted signals. Furthermore, this denoising method can effectively overcome the influence of external monitoring environments, further ensuring that microseismic monitoring technology can be applied across various domains, such as rock bursts, hydraulic fracturing monitoring, or mining safety.

## 2. Methods

### 2.1. Ensemble Empirical Mode Decomposition

Applying the empirical mode decomposition (EMD) to the microseismic recording adaptively produces a family of intrinsic mode functions (IMFs) in descending. The decomposed IMFs correspond to the different frequency compositions of the original signal [34]. However, this process involves a drawback of mode mixing, resulting in inaccurate IMF components, depriving the IMFs of their intrinsic single-scale features, and forming an oscillation in mixed scale [35]. To address this, Wu and Huang [36] developed the ensemble EMD (EEMD) method, which can effectively avoid mode aliasing using the noise-assisted approach. The establishment process of EEMD is as follows:

Step 1: Obtain the target signal $X_{i}\left(t\right)$ by mixing the original signal x(t) with the white Gaussian noise $w_{i}\left(t\right)$:

(1) $$X_{i}\left(t\right)=x\left(t\right)+\varepsilon w_{i}\left(t\right),i=1,2,\ldots ,M$$

where *M* represents the total of the mixed noise, and ε is its amplitude.

Step 2: Decompose the target signal $X_{i}\left(t\right)$ into a family of IMFs ($C_{ji}\left(t\right)$) employing EMD:

(2) $$X_{i}\left(t\right)=\sum_{j=1}^{n}C_{ji}\left(t\right)+r_{ni}\left(t\right)$$

where $r_{ni}\left(t\right)$ is the residual of $X_{i}\left(t\right)$, which is the residual of that the *n* IMFs are gained.

Step 3: Repeat the above two steps *M* times by mixing various noise sequences to the original data.

Step 4: The actual individual IMFs are obtained using the (ensemble) average values of the decomposed IMFs:

(3) $$C_{j}\left(t\right)=\frac{1}{M}\sum_{i=1}^{M}C_{ji}\left(t\right),j=1,2,\ldots ,n.$$

**Figure 1:**
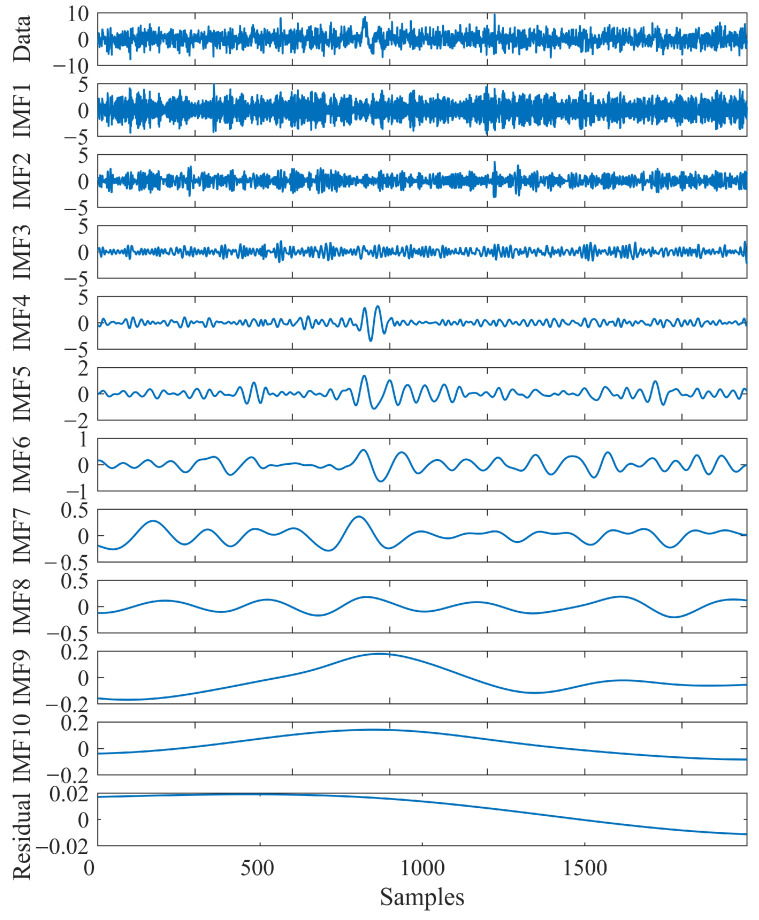
The decomposition result of the synthetic microseismic data using the EEMD method. The principal frequency of the synthetic P-wave is 100 Hz, and a sampling rate of 4 kHz using Rick wavelet, with an SNR of −15 dB.

As an effective signal decomposition method, EEMD is a typical application combing with the noise-assisted preprocessing method [37,38]. Considering the specialized procedure of EEMD, both a reasonable loop time and an appropriate amplitude of the added white noise are essential during the execution of EMD. Generally, to alleviate the impact of the added noise on the IMFs, the amplitude of the added noise should be minimized. However, if the amplitude of the added noise is too low, it will not produce extremal changes in the mixed data during critical steps of the EEMD computation [36]. Moreover, a proper ensemble counts yields reliable results, maintaining the error induced by the added noise at a sufficiently diminutive or even insignificant level [35]. Accordingly, adhering to Wu and Huang [36]’s recommendation, we selected an amplitude for the added white noise ε, approximately equal to 0.2 times the standard deviation of the decomposed data, and its ensemble counts *N* is 100. To verify the decomposition efficacy of the EEMD method in decomposing microseismic data, we established a synthetic microseismic recording with a signal-to-noise ratio (SNR) of −15 dB and adaptively decomposed it into 11 components. As shown in Figure 1, the decomposition result demonstrated that the decomposed IMFs sequentially correspond to different frequency compositions of the original signal in descending order. The distinguishable IMFs facilitate the extraction of useful signals from complex microseismic recordings.

After obtaining a series of IMFs employing the EEMD method, we need to determine those IMFs that are substantively useful for reconstructing new microseismic signals. This necessitates the delineation of a valid and reliable boundary to discriminate between noise and useful signals within noisy microseismic recordings. Our study employs Sample Entropy (SamEn) to facilitate this process.

### 2.2. Sample Entropy

The SamEn represents an improved measure method for time series complexity based on approximate entropy (ApEn) [39,40], and was widely applied to evaluate the complexity of time sequences, as well as in diverse domains such as medicine and economics. Notably, the SamEn algorithm is particularly suitable for judging the complexity of time-series signals, which means that the smaller the SamEn value, the higher self-similarity and the lower the noise [41]. In this investigation, the SamEn value is used to determine the randomness of microseismic signals, thereby facilitating the further distinguishing noise signals and microseismic events.

The procedures for calculating the SamEn value of the aforementioned IMFs are as follows:

Step 1: Define $C_{f}\left(i\right)$ as the obtained IMFs sequences using the EEMD algorithm, (i=1,2,…,N),f represents the total of IMFs.

Step 2: Construct a series of m-dimensional blocks using the $C_{f}\left(i\right)$:

(4) $$X\left(i\right)=C_{f}\left(i\right),C_{f}\left(i+1\right),\ldots ,C_{f}\left(i+m-1\right)$$

where i=1,2,…,k, and k=N−m+1.

Step 3: Calculate the distance between each sequence of the X(i) and all the *k* groups of X(j). This distance is defined as:

(5) $$d_{ij}=max\left|C_{f}\left(i+1\right)-C_{f}\left(j+l\right)\right|\left(i\neq j,l=0,1,2,\ldots ,m-1\right)$$

Step 4: Define T=r×SD, where *r* is set as 0.1–0.25, and SD represents the standard deviation of the calculated sequences. Then, count the number that the $d_{ij}$ is higher than *T* by row, noted as $B_{i}^{m}$. Define the $A_{i}^{m}$ as:

(6) $$\frac{1}{n-m}\times B_{i}^{m}.$$

The mean of all the $A_{i}^{m}$ is defined as:

(7) $$\phi^{m}=\frac{1}{n-m}\sum_{i=1}^{n-m}A_{i}^{m}$$

Step 5: m=m+1, repeat the steps 1–4.

Step 6: Define the sample entropy as:

(8) $$SamEn=ln\phi^{m}-ln\phi^{m+1}$$

**Figure 2:**
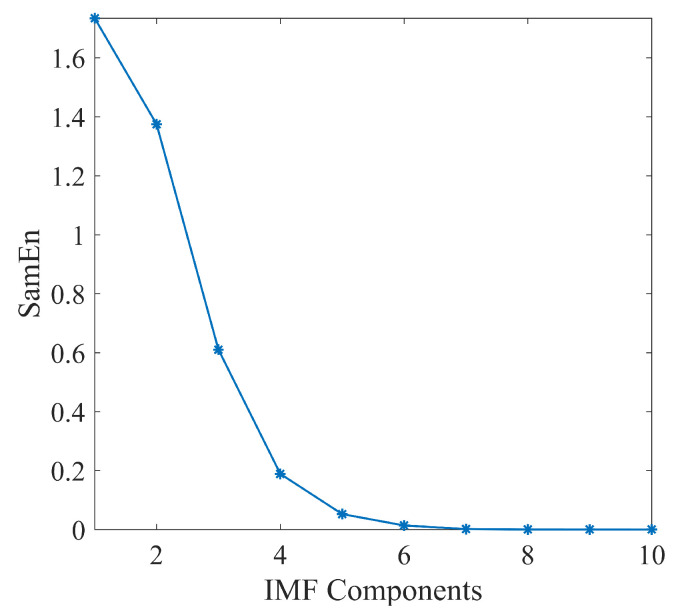
The SamEn value of the aforementioned decomposed IMFs.

From the definition of SampEn, it is evident that the *m* and *T* are two important factors in its computation. Guided by [42], established values of *m* are 1 or 2, while *T* is conventionally set between 0.1 and 0.25 times the standard deviation of the signal under consideration. Consequently, to distinguish the different characteristics of the decomposed IMFs, we computed the SampEn of the IMFs utilized in Figure 1, the corresponding parameters *m* is set as 1, and *T* is 0.15×std(IMF). The SampEn values for different IMFs are illustrated in Figure 2, revealing a distinguished decrease in SampEn values with ascending IMFs. In other words, this further reveals that the higher-order IMF components exhibit lower complexity and potentially contain less noise. These typical characteristics in Figure 2 may assist in extracting useful microseismic signals.

### 2.3. Microseismic Signal Extraction

Upon applying EEMD to microseismic recordings to obtain a series of IMFs, these IMFs are adaptively compiled in descending order based on the different frequency components of the decomposed signal. Subsequently, we compute the SampEn values of the different IMFs, illustrating their respective complexities. In order to extract the useful IMF components, a reasonable threshold needs to be determined to eliminate redundant IMF components. Empirically, IMF components with SampEn values exceeding 0.2 are discarded. Finally, by accumulating the residual IMF components, the ultimate microseismic signal is reconstructed.

**Figure 3:**
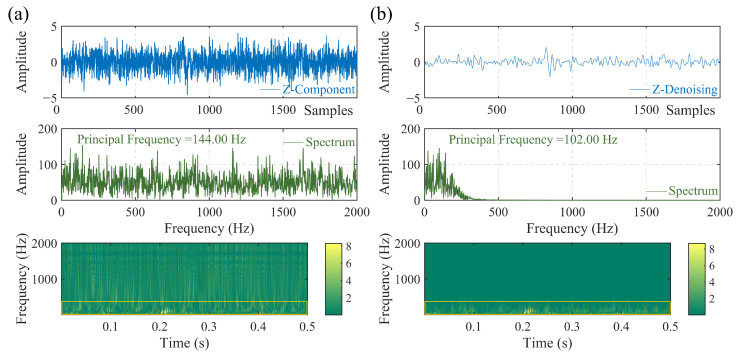
Comparison of Time-frequency analysis and spectrum analysis. (**a**) Synthetic noisy microseismic data with a principal frequency of 100 Hz and a sampling rate of 4 kHz using Rick wavelet, added with white Gaussian noise at an SNR of −15 dB. (**b**) The reconstructed microseismic data using the selected IMFs.

To further validate the capability of the developed signal extraction algorithm, we conducted spectral and time-frequency analyses. Figure 3 demonstrates that the principal frequency of the reconstructed data are consistent with the corresponding original data, and the principal frequency of the original data are preset with a stationary frequency while being synthesized. Moreover, the high-frequency information can be significantly eliminated while the low-frequency composition is reliably preserved. Additionally, the reconstructed data can avoid phase distortion (the P phase arrival point of the synthetic data are preset at 813). Consequently, the extracted data can be reasonably applied to verify onset times.

## 3. The Improved Picking Algorithm

In this study, we apply the AIC picker to estimate the arrival time of the extracted microseismic data. A robust AIC picker named M-AIC is employed to perform the AIC calculation, an approach developed by Maeda [7]. Furthermore, considering the picking speed, the AIC calculation time window (TW) should be determined to conserve the picking consumption. The specific picking procedures are as follows:

Step 1: Data extraction. Utilizing the EEMD and SamEn to decompose and reconstruct the noisy microseismic recording, thus extracting high-quality, useful microseismic signals.

Step 2: Segment selection of the M-AIC Picker. For clean microseismic recordings, the arrival time should be located prior to the peak signal amplitude. In this sense, and considering the computing consumption, the point of maximum signal amplitude is taken as the cutoff of the calculated data segment.

Step 3: Arrival time identification. The M-AIC picker is employed to the selected data segment of the extracted microseismic data using the algorithm as mentioned above, subsequently determining the first arrival at the minimum value within the obtained M-AIC sequences.

**Figure 4:**
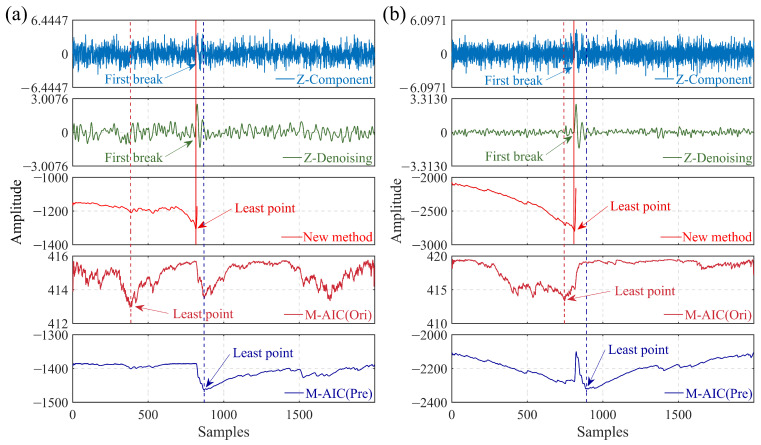
Arrival time estimation of different picking regulations employing the M-AIC picker for synthetic data. (Ori) represents the noisy synthetic signal, (Pre) is the denoised signal, the red dotted line represents the picking result of the M-AIC picker for the noisy signal, the red solid line represents the picking result of the proposed method, and the blue dotted line is the picking result of the M-AIC picker for the denoised signal. (**a**) Synthetic signal with white Gaussian noise (SNR = −15 dB). (**b**) Synthetic signal with colored (blue) noise (SNR = −15 dB).

To exhibit the efficacy of the developed picking method, the improved algorithm is applied to synthetic microseismic recordings that were integrated with different kinds of noise. Figure 4 illustrates the results obtained by executing the conventional M-AIC picker on the constrained calculation segment of the reconstructed signal, the entire denoised signal, and the original noisy signal, respectively. Evident from Figure 4, the reconstructed denoised signal can reliably substitute the original signal for the preliminary picking calculation. In other words, the proposed method can achieve accurate arrival time picking of microseismic recordings with high noise.

## 4. Results and Discussion

### 4.1. Picking Results Comparison of Different Approaches Using Synthetic Microseismic Recordings

**Figure 5:**
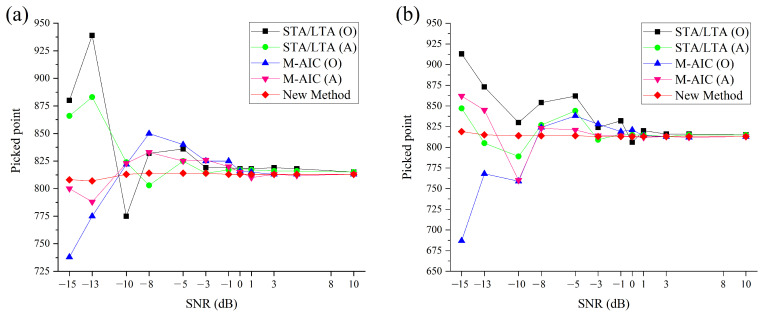
Arrival time evaluation results using different picking approaches for the noisy data. (O) means the original noisy signal, and (A) is the denoised signal. (**a**) Synthetic data added with white Gaussian noise. (**b**) Synthetic data added with colored (blue) noise.

In order to evaluate the validity of the improved method in this study, comparative tests were conducted utilizing different picking approaches, including the proposed algorithm, the popular M-AIC picker, and the conventional STA/LTA method. Akram and Eaton [43] provided a reasonable time window (TW) to choose the criterion for the STA/LTA method. In addition, the aforementioned approaches are applied to different synthetic microseismic recordings mixed with various noise, and the accumulated noise is given with different SNRs (SNRs are set between 10 dB and −15 dB). We design 100 distinct microseismic recordings using diverse noise at each SNR to derive average picking results of the abovementioned methods. Figure 5 demonstrates that the proposed algorithm can yield accurate and reliable picking results across varying SNRs for white and colored (blue) noise. However, SNR when the SNR is below −1 dB, traditional M-AIC, and STA/LTA pickers, despite de-noising the original signal, fail to obtain accurate arrival times. Specifically, the M-AIC picker exhibits more minor picking errors while being applied to the microseismic data, which has been de-noised utilizing the developed de-noising strategy. Furthermore, the STA/LTA picker is easier to involve in uncertain picking results since the TW length relies heavily on the frequency features of the microseismic data, which necessitates dynamic adjustments for different microseismic recordings [44].

**Figure 6:**
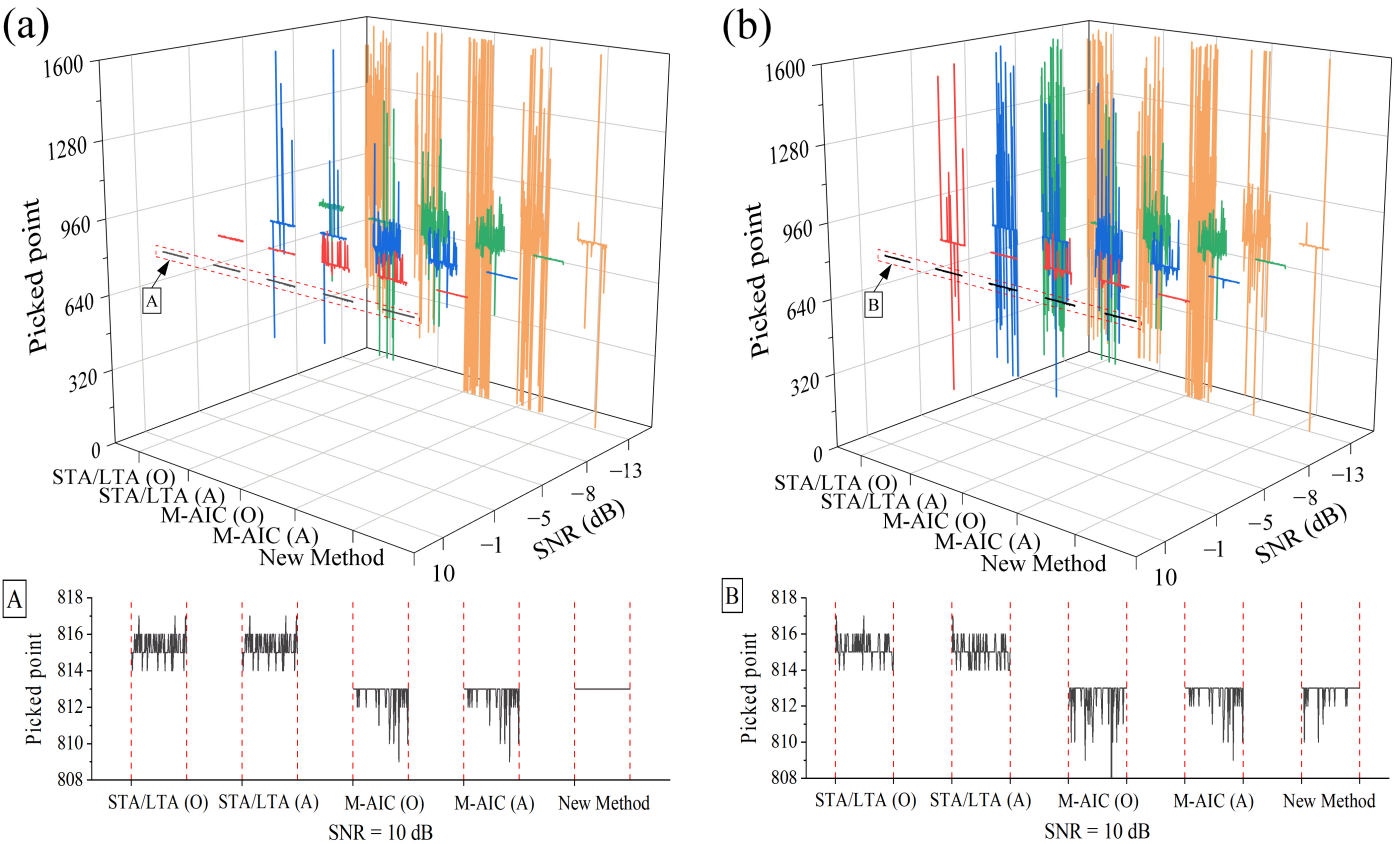
Stability comparison using different picking approaches for synthetic microseismic data with different SNRs, A and B under each subgraph are the corresponding enlarged picking results of the red dotted box. (O) means the original noisy signal, and (A) is the denoised signal. (**a**) Synthetic data added with white Gaussian noise. (**b**) Synthetic data added with colored (blue) noise.

In addition, stability represents an important indicator in evaluating the efficacy of the proposed picking method. Figure 6 is a three-dimension plot of statistical curves, derived from the computation results of 100 picking results using different picking approaches, based upon synthetic microseismic recordings at five distinct SNRs (with the first arrival point preset at 813). These results reveal that, compared to other popular pickers, the new picker can achieve more stable and accurate picking results at the designated SNRs of 10 dB, −1 dB, −5 dB, −8 dB, and −10 dB. Moreover, the new method has several random mutational picking results at −10 dB, possibly due to the effective microseismic signal and the added random noise having an approximate principal frequency. The overall trend of Figure 6 indicates that the picking results from the microseismic data with added colored (blue) noise fluctuate more than those from the microseismic data with added white Gaussian noise. In addition, when the SNR is set to 10 dB, the proposed picker does not fluctuate when the synthetic data are mixed with white Gaussian noise, but with the colored (blue) noise, the new picker has an error of 3 sampling intervals. This issue might be attributed to the frequency approximation between the useful microseismic event and the colored (blue) noise. Furthermore, while the STA/LTA algorithm can maintain better stability compared to the M-AIC algorithm, its picking accuracy is not as good as the latter.

### 4.2. Application on Real Data

We conducted comparative tests on real microseismic data, thereby further confirming the effectiveness of the new method in estimating the arrival time of microseismic recordings. The real microseismic recordings were collected from a deeply buried tunnel excavation monitoring project. The configuration of microseismic monitoring consists of two rows spaced about 10 m apart with eight accelerometers, which were deployed on the sidewalls of the tunnel. The sample rate of the microseismic monitoring system was set to 6000 Hz.

**Figure 7:**
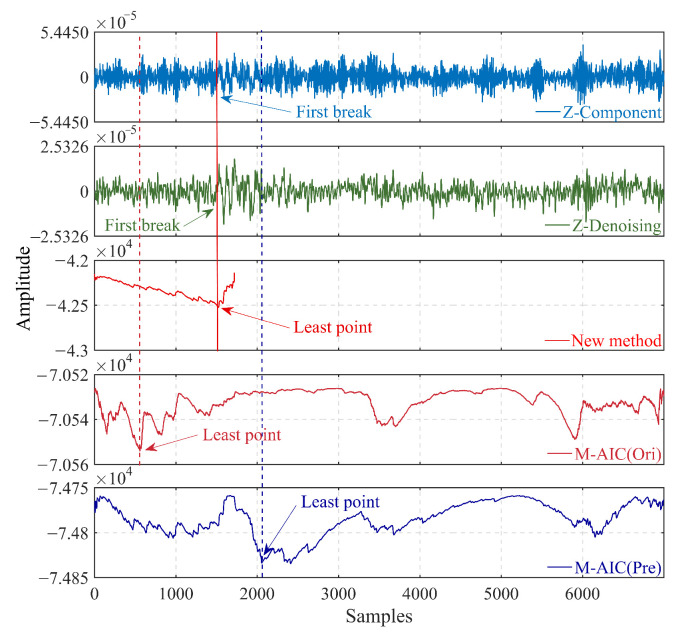
Arrival time identification results of different picking regulations for the real microseismic recording. The red solid line represents the picking result of the proposed method.

The comparison process involves applying the proposed algorithm and the M-AIC method directly to the original and denoised microseismic data. As shown in Figure 7, several intuitive microseismic events are displayed in the original Z component, which can serve as useful location events. However, the denoising results from Z-denoising show several random pseudo-microseismic events, which might originate from field environmental noise. In summary, the proposed approach can achieve reliable first arrival identification. In contrast, we cannot determine a reasonable arrival time when the M-AIC method is applied to the original microseismic recordings.

To evaluate the computational time consumption of the new method, we apply different picking approaches to process the same microseismic recording. The recording contains 1600 sampling points, and the test was conducted on Windows 10 with 8 GB RAM and a 3.5 GHz processor. The results show that the time required for the new method, M-AIC (A), STA/LTA (A), M-AIC (O), STA/LTA (O) are 12.81 s,12.79 s, 12.83 s, 0.046 s, and 0.02 s, respectively. Clearly, picking the first arrival after denoising consumes more time than the picking process of original microseismic data because the denoising process requires a significant amount of computational time. Notably, sacrificing time consumption to enhance the accuracy of first arrival estimation might be an acceptable method, especially for the effective microseismic signal extraction using EEMD and SamEn. Furthermore, considering the performance of advanced computational terminals, time cost may not restrict the application of the proposed automatic arrival time estimation method.

In addition, to validate the effectiveness of the proposed method, we conducted a comparative test using the abovementioned real microseismic recording, comparing traditional finite impulse response (FIR) filter (based on the Gauss window with 21 orders, and the cut-off frequency is set as 100 Hz), wavelet hard threshold denoising (using the heuristic thresholding rule ’heursure’ rule, db4 wavelet basis, and the decomposition level is 3), and the proposed strategy. Comparison results demonstrate that the proposed approach can achieve a better denoising result, a higher signal-to-noise ratio (SNR), and a clear first arrival. The SNR for the original microseismic recording, the denoised signal of the traditional FIR filter, the denoised signal of the wavelet hard threshold denoising method, and the denoised signal of the proposed strategy are 0.0013782 dB, 0.0093141 dB, 0.0054686 dB, and 0.016775 dB, respectively. When calculating the SNR, the mean square values are used to estimate signal and noise power.

## 5. Conclusions

This paper presents an effective method for estimating the arrival time of high-noise microseismic data, aiming to alleviate the influences of strong noise and data segment selection. Combined with the characteristics of microseismic recordings, the microseismic signal is subjected to EEMD, yielding a series of IMFs. Subsequently, we utilize the threshold determined by the SamEn values of different IMFs to select several useful IMF components. IMF components with SamEn values greater than 0.2 are removed. The selected IMF components are then reconstructed into the final microseismic signal, which is used to construct the picking target signal. Ultimately, the M-AIC algorithm is employed to estimate the arrival time of the constrained computation data segment, which is determined by setting the maximum point of the reconstructed signal amplitude.

Our results suggest that the new strategy can keep the time-frequency features of the original recordings, and the denoised results illustrate that the extracted data can be used for subsequent arrival time estimation. When applying the new method to synthetic microseismic data with different SNRs, the proposed approach can accomplish picking errors within 1–3 sampling intervals. Additionally, the proposed picker maintains higher stability when employed for synthetic microseismic data with various SNRs compared to other popular pickers. Furthermore, applications in real microseismic recordings further reveal the effectiveness and reliability of the new strategy.

## Data Availability

This code and data can be obtained from the Github repository, which is located at https://github.com/lhl-cdut/Microseismic-arrival-time-picking, accessed on 21 November 2023.

## References

[1] Tang S., Li J., Tang L., Zhang L. (2023). Microseismic monitoring and experimental study on rockburst in water-rich area of tunnel. Tunn. Undergr. Space Technol..

[2] He J., Li H., Tuo X., Wen X., Feng L. (2023). A Reliable Online Dictionary Learning Denoising Strategy for Noisy Microseismic Data. IEEE Trans. Geosci. Remote Sens..

[3] Dong L., Zhu H., Yan F., Bi S. (2023). Risk field of rock instability using microseismic monitoring data in deep mining. Sensors.

[4] Saad O.M., Shalaby A., Samy L., Sayed M.S. (2018). Automatic arrival time detection for earthquakes based on Modified Laplacian of Gaussian filter. Comput. Geosci..

[5] Li H., Tuo X., Shen T., Wang R., Courtois J., Yan M. (2017). A new first break picking for three-component VSP data using gesture sensor and polarization analysis. Sensors.

[6] Long Y., Lin J., Li B., Wang H., Chen Z. (2019). Fast-AIC Method for Automatic First Arrivals Picking of Microseismic Event With Multitrace Energy Stacking Envelope Summation. IEEE Geosci. Remote Sens. Lett..

[7] Maeda N. (1985). A method for reading and checking phase times in autoprocessing system of seismic wave data. Zisin.

[8] Akaike H. (1971). Autoregressive model fitting for control. Ann. Inst. Stat. Math..

[9] Akaike H. (1974). Markovian representation of stochastic processes and its application to the analysis of autoregressive moving average processes. Ann. Inst. Stat. Math..

[10] Allen R.V. (1978). Automatic earthquake recognition and timing from single traces. Bull. Seismol. Soc. Am..

[11] Han L., Wong J., Bancroft J. (2009). Time picking and random noise reduction on microseismic data. CREWES Res. Rep..

[12] Wu H., Zhang B., Li F., Liu N. (2019). Semiautomatic first-arrival picking of microseismic events by using the pixel-wise convolutional image segmentation method. Geophysics.

[13] Bao Y., Jia J. (2019). Improved time-of-flight estimation method for acoustic tomography system. IEEE Trans. Instrum. Meas..

[14] Coppens F. (1985). First arrival picking on common-offset trace collections for automatic estimation of static corrections. Geophys. Prospect..

[15] Küperkoch L., Meier T., Lee J., Friederich W., Group E.W. (2010). Automated determination of P-phase arrival times at regional and local distances using higher order statistics. Geophys. J. Int..

[16] Shang X., Li X., Morales-Esteban A., Dong L. (2018). An improved p-phase arrival picking method S/LKA with an application to the Yongshaba mine in China. Pure Appl. Geophys..

[17] Kalkan E. (2016). An automatic P-phase arrival-time picker. Bull. Seismol. Soc. Am..

[18] Ross Z.E., Ben-Zion Y. (2014). Automatic picking of direct P, S seismic phases and fault zone head waves. Geophys. J. Int..

[19] Li H., Tuo X., Wang R., Courtois J. (2019). A Reliable Strategy for Improving Automatic First-Arrival Picking of High-Noise Three-Component Microseismic Data. Seismol. Res. Lett..

[20] Gaci S. (2013). The use of wavelet-based denoising techniques to enhance the first-arrival picking on seismic traces. IEEE Trans. Geosci. Remote Sens..

[21] Tsai K.C., Hu W., Wu X., Chen J., Han Z. (2019). Automatic First Arrival Picking via Deep Learning With Human Interactive Learning. IEEE Trans. Geosci. Remote Sens..

[22] Ross Z.E., Meier M.A., Hauksson E. (2018). P wave arrival picking and first-motion polarity determination with deep learning. J. Geophys. Res. Solid Earth.

[23] Guo C., Zhu T., Gao Y., Wu S., Sun J. (2021). AEnet: Automatic picking of P-wave first arrivals using deep learning. IEEE Trans. Geosci. Remote Sens..

[24] Bose S., De A., Chakrabarti I. (2021). Area-delay-power efficient VLSI architecture of FIR filter for processing seismic signal. IEEE Trans. Circuits Syst. II Express Briefs.

[25] Suman S., Kumar A., Singh G.K. (2016). A new method for higher-order linear phase FIR digital filter using shifted Chebyshev polynomials. Signal Image Video Process..

[26] Nasr M., Giroux B., Dupuis J.C. (2021). A novel time-domain polarization filter based on a correlation matrix analysis. Geophysics.

[27] Li L., Li H., Tuo X., Yang Z., Rong W. (2023). A Novel Polarization Estimation Method for Seismic Recordings. Seismol. Soc. Am..

[28] Li H., Shi J., Li L., Tuo X., Qu K., Rong W. (2022). Novel wavelet threshold denoising method to highlight the first break of noisy microseismic recordings. IEEE Trans. Geosci. Remote Sens..

[29] Du C., Yu S., Yin H., Sun Z. (2022). Microseismic time delay estimation method based on continuous wavelet. Sensors.

[30] Zhang C., van der Baan M. (2019). Microseismic denoising and reconstruction by unsupervised machine learning. IEEE Geosci. Remote Sens. Lett..

[31] Chen Y., Saad O.M., Savvaidis A., Chen Y., Fomel S. (2022). 3D microseismic monitoring using machine learning. J. Geophys. Res. Solid Earth.

[32] Mousavi S.M., Beroza G.C. (2022). Deep-learning seismology. Science.

[33] Saad O.M., Bai M., Chen Y. (2021). Uncovering the microseismic signals from noisy data for high-fidelity 3D source-location imaging using deep learning. Geophysics.

[34] Huang N.E., Shen Z., Long S.R., Wu M.C., Shih H.H., Zheng Q., Yen N.C., Tung C.C., Liu H.H. (1998). The empirical mode decomposition and the Hilbert spectrum for nonlinear and non-stationary time series analysis. Proc. R. Soc. L. Ser. Math. Phys. Eng. Sci..

[35] Huang N.E., Wu Z. (2008). A review on Hilbert-Huang transform: Method and its applications to geophysical studies. Rev. Geophys..

[36] Wu Z., Huang N.E. (2009). Ensemble empirical mode decomposition: A noise-assisted data analysis method. Adv. Adapt. Data Anal..

[37] Zhao Y., Zhong Z., Li Y., Shao D., Wu Y. (2023). Ensemble empirical mode decomposition and stacking model for filtering borehole distributed acoustic sensing records. Geophysics.

[38] Wang Y., Satake K., Maeda T., Shinohara M., Sakai S. (2020). A method of real-time tsunami detection using ensemble empirical mode decomposition. Seismol. Res. Lett..

[39] Richman J.S., Moorman J.R. (2000). Physiological time-series analysis using approximate entropy and sample entropy. Am. J. Physiol.-Heart Circ. Physiol..

[40] Delgado-Bonal A., Marshak A. (2019). Approximate entropy and sample entropy: A comprehensive tutorial. Entropy.

[41] Zhou X., Lei W. (2020). Spatial patterns of sample entropy based on daily precipitation time series in China and their implications for land surface hydrological interactions. Int. J. Climatol..

[42] Pincus S.M., Goldberger A.L. (1994). Physiological time-series analysis: What does regularity quantify?. Am. J. Physiol.-Heart Circ. Physiol..

[43] Akram J., Eaton D.W. (2016). A review and appraisal of arrival-time picking methods for downhole microseismic dataArrival-time picking methods. Geophysics.

[44] Zhang Y., Chen Q., Liu X., Zhao J., Xu Q., Yang Y., Liu G. (2018). Adaptive and automatic P-and S-phase pickers based on frequency spectrum variation of sliding time windows. Geophys. J. Int..

